# Steric and Electronic Effect of Cp-Substituents on the Structure of the Ruthenocene Based Pincer Palladium Borohydrides

**DOI:** 10.3390/molecules25092236

**Published:** 2020-05-09

**Authors:** Sergey V. Safronov, Elena S. Osipova, Yulia V. Nelyubina, Oleg A. Filippov, Irina G. Barakovskaya, Natalia V. Belkova, Elena S. Shubina

**Affiliations:** A. N. Nesmeyanov Institute of Organoelement Compounds, Russian Academy of Sciences, 28 Vavilova Str., Moscow 119991, Russia; aosipova92@gmail.com (E.S.O.); unelya@xrlab.ineos.ac.ru (Y.V.N.); h-bond@ineos.ac.ru (O.A.F.); analyst@ineos.ac.ru (I.G.B.); shu@ineos.ac.ru (E.S.S.)

**Keywords:** pincer, ligand, palladium, ruthenocene, tetrahydroborate

## Abstract

Ruthenocene-based PCP^tBu^ pincer ligands were used to synthesize novel pincer palladium chloride Rc^F^[PCP^tBu^]PdCl (**2a**) and two novel palladium tetrahydroborates Rc^F^[PCP^tBu^]Pd(BH_4_) (**3a**) and Rc^*^[PCP^tBu^]Pd(BH_4_) (**3b**), where Rc^F^[PCP^tBu^] = κ^3^-{2,5-(tBu_2_PCH_2_)_2-_C_5_H_2_}Ru(Cp^F^) (Cp^F^ = C_5_Me_4_CF_3_), and Rc*[PCP^tBu^] = κ^3^-{2,5-(tBu_2_PCH_2_)_2_C_5_H_2_}Ru(Cp*) (Cp* = C_5_Me_5_). These coordination compounds were characterized by X-ray, NMR and FTIR techniques. Analysis of the X-ray data shows that an increase of the steric bulk of non-metalated cyclopentadienyl ring in **3a** and **3b** relative to non-substituted Rc[PCP^tBu^]Pd(BH_4_) analogue (**3c**; where Rc[PCP^tBu^] = κ^3^-{2,5-(tBu_2_PCH_2_)_2_C_5_H_2_}Ru(Cp), Cp = C_5_H_5_) pushes palladium atom from the middle plane of the metalated Cp ring in the direction opposite to the ruthenium atom. This displacement increases in the order **3c** < **3b** < **3a** following the order of the Cp-ring steric volume increase. The analysis of both X-ray and IR data suggests that BH_4_ ligand in both palladium tetrahydroborates **3a** and **3b** has the mixed coordination mode η^1,2^. The strength of the BH_4_ bond with palladium atom increases in the order Rc[PCP^tBu^]Pd(BH_4_) < Rc*[PCP^tBu^]Pd(BH_4_) < Rc^F^[PCP^tBu^]Pd(BH_4_) that appears to be affected by both steric and electronic properties of the ruthenocene moiety.

## 1. Introduction

**Scheme 1:**
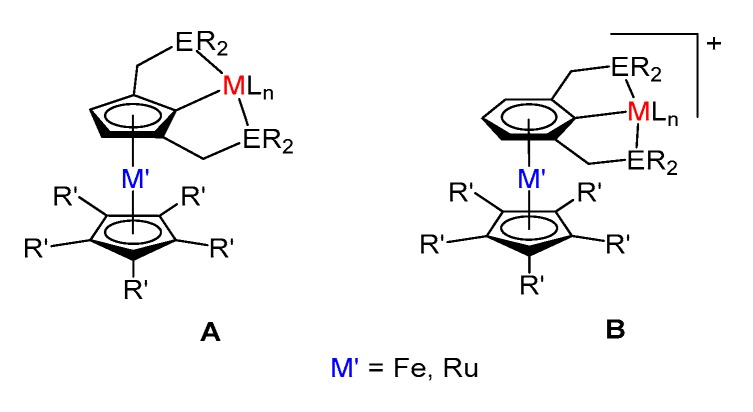
General structures of metallocene-based pincer complexes. ER_2_ = PR_2_, NR_2_ where R = alkyl, aryl; R’ = H, CH_3_, CF_3_, etc.

The chemistry of transition metal complexes with pincer-type ligands has been actively studied since the 1970s [1]. Such complexes feature a unique balance of stability and reactivity, which can be controlled by the systematic modification of ligands, and thus a huge potential for the use in organic synthesis and catalysis [2,3,4,5,6,7,8,9,10,11,12]. A continuous search for more active and selective catalysts, including asymmetric ones, has led to the appearance of new bimetallic complexes of transition metals with pincer ligands of sandwich-type containing cyclometalated five- [13,14,15,16,17,18,19,20,21,22] (**A**) and six-membered [23,24,25,26,27,28,29,30] (**B**) aromatic rings (Scheme 1). 

The presence of a transition metal in the sandwich moiety allows varying the charge of the bimetallic complex and significantly affects the electrochemical characteristics of the chelated η^1^-metal center [23]. For example, in comparison with neutral precursors, the presence of a positive charge in bimetallic ruthenium–palladium complexes leads to a decrease in the electron density on the chelated palladium atom and contributes to an increase in its catalytic activity in Suzuki cross-coupling reactions [19,24,26]. It can be assumed that the introduction of electron-withdrawing groups into the sandwich core of the bimetallic complex can favorably affect the catalytic properties of the chelated metal atom. On the other hand, it is well known that in addition to the electronic factor, the catalytic activity of metal complexes is strongly affected by steric factors determining the spatial availability of the metal center for the substrate [8,15,16,31]. In the case of bimetallic PCP complexes of sandwich-type, the steric accessibility of the chelated metal atom is determined not only by the volume of organic groups R at the phosphorus donor atoms and the P-M-P angle but also by the presence of substituents in the non-metalated ring [17,23,25]. Thus, the presence of five methyl groups in the cyclopentadienyl ligand significantly increases its steric volume and donor ability in comparison with the unsubstituted Cp ring (Cp = η^5^-C_5_H_5_). Replacing one of the methyl groups in the Cp* ligand (Cp* = η^5^-C_5_Me_5_) with CF_3_ group (Cp^F^ = C_5_Me_4_CF_3_) slightly increases the volume of the five-membered ring, but keeps the electronic effect of the ligand at the level of unsubstituted cyclopentadienyl [32,33,34,35]. 

An interesting feature of palladium chloride complexes based on ferrocene and ruthenocene is their ability to react with NaBH_4_ to form the corresponding tetrahydroborate complexes Fc[PCP^tBu^]Pd(BH_4_) and Rc[PCP^tBu^]Pd(BH_4_). (where Fc[PCP^tBu^] and Rc[PCP^tBu^] = κ^3^-{2,5-(^t^Bu_2_PCH_2_)_2_C_5_H_2_}M(Cp), M = Fe, Ru) [15,36]. Rc[PCP^tBu^]Pd(BH_4_) complex, which we have recently studied [36], proved to be a useful starting compound for the production of highly reactive palladium hydride Rc[PCP^tBu^]PdH. However, despite a high potential of complexes of this type, the sterically loaded palladium pincer complexes remain almost unexplored. In this work, we report on the synthesis of two new palladium borohydride pincer complexes based on substituted ruthenocenes. The use of Cp* and Cp^F^ ligands and comparison with the non-substituted Cp analogues allows accessing the steric and electronic effects of sandwich moiety on the properties of PCP^tBu^ chelated palladium. 

## 2. Results and Discussion

### 2.1. Synthesis

**Scheme 2:**
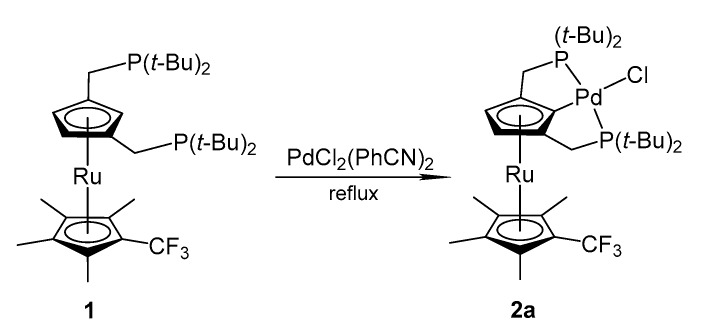
Cyclopalladation reaction.

1,3-disubstituted ruthenocene bisphosphine with a Cp^F^ ligand in the metallocene core {1,3-(^t^Bu_2_PCH_2_)_2_C_5_H_2_}Ru(Cp^F^) (**1**) was synthesized by phosphination of the corresponding diol {1,3-(HOCH_2_)_2_C_5_H_2_}Ru(Cp^F^) in acetic acid according to the previously published procedure [37]. The cyclometalation of **1** with PdCl_2_(PhCN)_2_ in 2-methoxyethanol under reflux for 3 h (Scheme 2) yields the new palladium complex Rc^F^[PCP^tBu^]PdCl (**2a**) (where Rc^F^[PCP^tBu^] = κ^3^-{2,5-(tBu_2_PCH_2_)_2_C_5_H_2_}Ru(C_5_Me_4_CF_3_)).

As the reaction proceeds, a finely dispersed, unidentified, bright yellow precipitate and impurities are formed that significantly complicate the subsequent isolation of the target cyclometalation product. To facilitate the isolation of **2a**, two equivalents of triethylamine were introduced into the reaction mixture after 3 h of reflux, which was continued for another 2 h. This approach led to the formation of a significant amount of palladium black, however, it allowed simplifying the purification procedure and gave the product with 31% yield. Note that the analogues palladium complex based on pentamethylruthenocene Rc^*^[PCP^tBu^]PdCl (**2b**) (where Rc^*^[PCP^tBu^] = κ^3^-{2,5-(^t^Bu_2_PCH_2_)_2_C_5_H_2_}Ru(C_5_Me_5_)) was previously obtained under similar conditions with 30% yield. Apparently, the serious steric hindrance in the bisphosphine molecule created by the presence of five substituents in the cyclopentadienyl ring and tert-butyl groups at the donor phosphorus atoms hampers the cyclometalation reaction. For comparison, [PCP^tBu^] complexes of palladium based on ferrocene, ruthenocene, and benzene can be obtained under comparable conditions with much higher yields [1,15,17]. It has been reported that in the preparation of benzene-based [POCOP^tBu^] palladium complex the replacement of the most frequently used PdCl_2_(PhCN)_2_ as a cyclometalating agent with palladium(II) chloride leads to an increase in the yield of the reaction product from 31 to 80% [38]. Unfortunately, the use of PdCl_2_ as a cyclometalation reagent in the reaction with Rc^F^[PCP^tBu^] (**1**) led to the formation of complex **2a** only in trace amounts. Compound **2a** is a pale yellow, air-stable powder. It was characterized by multinuclear NMR spectroscopy, and its purity was confirmed by elemental analysis.

The ^31^P{^1^H} and ^19^F NMR spectra of complex **2a** contain a single signal from two equivalent phosphorus nuclei and three equivalent fluorine nuclei at δ 82.07 ppm and −52.74 ppm, respectively. In the ^1^H NMR, the proton signal of the metallated cyclopentadienyl ring is observed as a singlet at 4.22 ppm (2H). The signal of the six protons of two methyl groups located at the 2′,5′ positions of the Cp^F^ ligand appears as a broadened multiplet at δ 1.89 ppm and the proton signals of two other methyl groups of the same ligand as a singlet at δ 1.77 ppm. Geminal methylene protons of CH_2_P^t^Bu_2_ groups are not equivalent and appear as doublets of virtual triplets at δ 2.45 and 2.51 ppm. Resonances of methyl protons of nonequivalent tert-butyl groups are observed in the form of two virtual triplets at δ 1.33 ppm and 1.47 ppm in accordance with the expected structure.

**Scheme 3:**
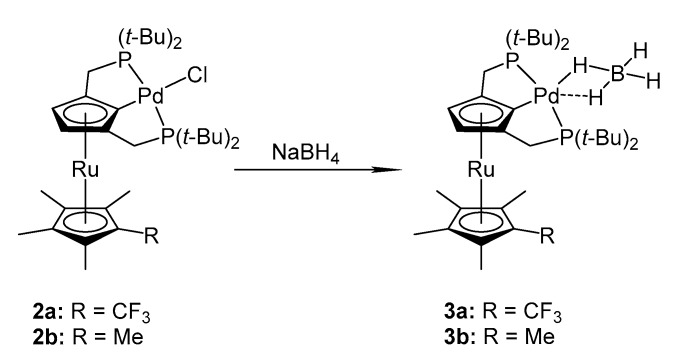
Synthesis of borohydrides **3a** and **3b**.

The reaction of Rc^F^[PCP^tBu^]PdCl (**2a**) and Rc^*^[PCP^tBu^]PdCl (**2b**) with an excess of NaBH_4_ in refluxing ethanol leads to the formation of the corresponding tetrahydroborate complexes Rc^F^[PCP^tBu^]Pd(BH_4_) (**3a**) and Rc^*^[PCP^tBu^]Pd(BH_4_) (**3b**) (Scheme 3).

The complete conversion of the starting chlorides **2** into borohydrides **3** can be reached in 8 h, adding extra NaBH_4_ to the reaction mixture every hour. Monitoring of the formation of complex **3b** using NMR spectroscopy showed that after 3 h of reflux the ratio of the reaction product to the starting compound is approximately 3:1. The formation of palladium hydride complexes was not observed. For comparison, the pincer chloropalladium complexes based on ferrocene Fc[PCP^tBu^]PdCl [15] and ruthenocene Rc[PCP^tBu^]PdCl [36] are completely converted to the corresponding tetrahydroborates in 2 h. Complexes **3a** and **3b** were isolated in analytically pure form with 90% and 93% yield, respectively, as bright yellow crystalline powders. They are stable at room temperature in the solid state but are sensitive to air and thermally unstable in solution. In chloroform, **3a** and **3b** rapidly transform into the chloropalladium precursors **2a** and **2b**. In general, sterically loaded borohydride complexes **3a** and **3b** are less stable than the previously obtained ruthenocene analogue Rc[PCP^tBu^]Pd(BH_4_) (**3c**) [36]. Compounds **3a** and **3b** were fully characterized by NMR, IR, and elemental analysis. The presence of BH_4_ ligand coordinated to the palladium atom is confirmed by ^1^H and ^11^B NMR spectroscopy. The BH_4_ protons appear in the ^1^H NMR spectrum as a strongly broadened multiplet at δ_H_ 0.13 and 0.21 ppm for **3a** and **3b** in C_6_D_6_, respectively. ^11^B{^1^H} NMR spectra exhibit broadened signals at δ −34.54 and −34.73, respectively. The broadening of the BH_4_ group signals in the ^1^H NMR spectra indicates a rapid averaging in solution of hydrogen atoms bound to boron. The ^31^P{^1^H} NMR spectra of complexes **3a** and **3b** exhibit singlets at δ_P_ 87.44 and 87.07, respectively, indicating the equivalence of two phosphorus nuclei.

### 2.2. X-Ray Diffraction Study

**Figure 1:**
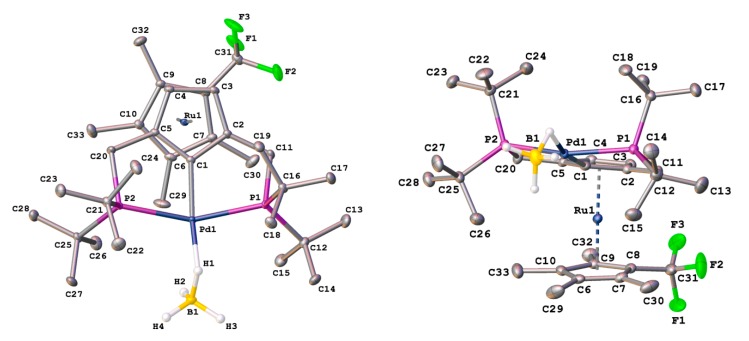
Two views of the compound **3a** with thermal ellipsoids at 50% probability level. Selected bond lengths (Å) and angles (°): Pd(1)–C(1) 1.9844(18), Pd(1)–P(1) 2.3447(5), Pd(1)–P(2) 2.3679(5), Pd(1)⋯B(1) 2.531(2), Pd(1)–H(1) 1.86(2), Pd(1)⋯H(2) 2.46(2); C(1)–Pd(1)–H(1) 167.9(6), Pd(1)–H(1)–B(1) 110.2(12), Pd(1)–H(2)–B(1) 80.2(12), C(1)–Pd(1)–B(1) 165.62(8), P(1)-Pd(1)-P(2) 155.67(2).

**Figure 2:**
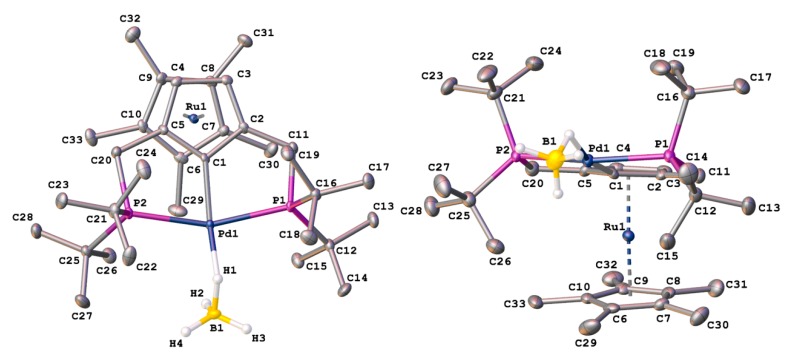
Two views of the compound **3b** in representation of atoms via thermal ellipsoids at 50% probability level. Selected bond lengths (Å) and angles (°): Pd(1)–C(1) 1.990(5), Pd(1)–P(1) 2.3409(13), Pd(1)–P(2) 2.3453(13), Pd(1)⋯B(1) 2.560(7), Pd(1)–H(1) 1.82(5), Pd(1)⋯H(2) 2.34(6); C(1)–Pd(1)–H(1) 166.8(18), Pd(1)–H(1)–B(1) 118(4), Pd(1)–H(2)–B(1) 90(3), C(1)–Pd(1)–B(1) 170.0(2), P(1)Pd(1)P(2) 158.87(5).

The structures of complexes **3a** and **3b** are very similar as confirmed by single-crystal XRD analysis (Figure 1 and Figure 2). They feature palladium atoms in a distorted square-planar environment with an angle P(1)-Pd(1)-P(2) equal to 155.67(2) and 158.87(5)°, respectively. Palladium atom deviates from the middle plane of the metalated Cp ring in the direction opposite to the ruthenium atom by 0.541(3) and 0.489(9) Å, respectively. For comparison, in the Rc[PCP^tBu^]Pd(BH_4_) complex (**3c**) the distance between the palladium atom and the plane of the Cp ring is 0.322 Å [36]. In both structures, the phosphorus atoms P(1) and P(2) rise above the Cp ring plane to different degrees: by 0.888(3) and 0.591(3) Å in case of **3a**; 0.708(10) and 0.501(11) Å in case of **3b**. The presence of five organic substituents in the non-metalated Cp ring makes the cyclopentadienyl rings noticeably non-coplanar. The angle between the planes of the cyclopentadienyl rings is 8.75(8) and 9.9(2)° in **3a** and **3b**, respectively. Similar values of this angle are reported for palladium chloride complex Rc*[PCP^tBu^]PdCl (**2b**) [17].

It is known that the BH_4_ anion can be coordinated to a transition metal atom in a monodentate (η^1^), bidentate (η^2^), or tridentate (η^3^) fashion [39]. According to our X-ray diffraction data, in complexes **3a** and **3b** the BH_4_ group is located in the *syn* position to the metallocene core relative to the Pd-C(1) bond line. The distance Pd-H(1) is 1.86(2) and 1.82(5) Å, respectively. These values significantly exceed the Pd-H bond length (1.53–1.75 Å) in the known palladium hydride pincer complexes [38,40,41] and are comparable to the values found for the ruthenocene-based complex Rc[PCP^tBu^]Pd(BH_4_) **3c** (1.87(2) Å) [36]. The distance of the Pd atom to the second nearest hydrogen atom H(2) in structures **3a**, **3b** is 2.46(2) and 2.34(6) Å, respectively (for comparison, d(Pd-H(2)) = 2.54(3) Å in **3c** [36]) that is below the sum of Pd-H van der Waals radii. The difference between the distances Pd-H(1) and Pd-H(2) in **3a** and **3b** Δd(Pd···H) = 0.60(2) and 0.52(5) Å is slightly lower than in **3c**. These values are comparable to Δd(Pd···H) in the previously described palladium borohydride complexes and fall in the reported range 0.2–0.67 Å [15,36,42,43]. An analysis of the above data votes for the monodentate η^1^ type of BH_4_ binding to palladium atom with more pronounced secondary Pd···H interaction relative to **3c**. However, according to our DFT calculations for complex **3c** the elongated Pd-H(2) contact makes an important additional contribution to the total binding of the BH_4_ fragment to the palladium atom [36]. We believe that in complexes **3a** and **3b**, featuring even shorter Pd-H(2) distances, the coordination of the BH_4_ ligand to the palladium atom can be described as the intermediate η^1,2^ type similar to **3c**.

In complexes **3a** and **3b**, the Pd···B distance exceeds the sum of the covalent radii of Pd and B atoms (ca. 2.2 Å) being 2.531(2) and 2.560 (7) Å, respectively. For comparison, in complex **3c**, the Pd···B distance is 2.587(3) Å [36]. Obviously, a decrease in the Pd···B distance in the series of compounds **3** indicates an increase in the strength of the BH_4_ bond with the palladium atom. For comparison, in the recently described monodentate and bidentate borohydride complexes supported by benzene based pincer ligands, the Pd···B distance varies in the range 2.42–2.47 Å [42,43]. It is interesting to note that the angle C(1)-Pd(1)-B(1) is 170.3(1)° in the complex Rc[PCP^tBu^]Pd(BH_4_) (**3c**) [36] and 170.0(2)° in Rc*[PCP^tBu^]Pd(BH_4_) (**3b**), while in Rc^F^[PCP^tBu^]Pd(BH_4_) (**3a**) it is 165.62(8)°. Presumably, the additional (relative to complexes **3b** and **3c**) deviation of 4.4–4.7° from linearity in **3a** is due to the greater steric pressure of the bulky axial *tert*-butyl groups at phosphorus atoms on the borohydride ligand. Indeed, the distance between the central carbon atoms C(16) and C(21) of the axial *tert*-butyl groups in complexes **3a** and **3b** is 5.573(3) and 5.759(6) Å, respectively. For comparison, the same distance in complex **3c** is 6.006(3) Å [36]. Thus, despite the bulky CF_3_ group is in the remote position relative to Pd(BH_4_) fragment, it affects the overall geometry of this pincer complex: the decreased inclination of two Cp rings puts three methyl groups close to *tert*-butyls pushing both tBu_2_P fragments above the plane of cyclometalated ring away from to the ruthenium atom. That, in turn, expels BH_4_ moiety below the same plane decreasing the C(1)-Pd(1)-B(1) angle.

### 2.3. IR Spectra

Vibrational spectra are often used to determine the type of BH_4_ group coordination to transition metal [39,44]. The IR spectra of complexes **3a** and **3b** are similar to the spectrum of the previously published Rc[PCP^tBu^]Pd(BH_4_) (**3c**) complex (Figures S14–S17). In agreement with the crystallographic data, they indicate the presence of a secondary Pd···H interaction in addition to the primary Pd-H bond both in the crystalline state and in solution. In the spectra of solid samples of complexes **3a** and **3b**, the stretching vibration ν^as^(B-H_term_) of the terminal B-H bonds is observed in the frequency range 2363–2388 cm^−1^ either as a split band (in case of **3a**) or as a band with a shoulder (**3b**). Probably, the splitting is associated with the isotopic effect of ^10^B-H/^11^B-H [39] vibrations. In the CH_2_Cl_2_ solution of complexes **3a** and **3b**, the same vibration appears as a non-split strong band at 2376 and 2372 cm^−1^, respectively. Note that, in the series of complexes **3**, a shift of this band to a higher frequency region is observed (Table 1), which may indicate an increase in the contribution of the bidentate form of BH_4_ coordination [39,44]. The stretching bands ν^s^(B-H_term_) of moderate intensity are observed in the IR spectrum of solid samples **3a** and **3b** at 2296 and 2291 cm^−1^, respectively. The stretching vibrations ν(B-H_bridge_) of a bridging hydrogen atom Pd-H-B in the spectra of complexes **3a**, **3b** appear as wide bands of moderate intensity at 2019 and 2025 cm^−1^ and a high-intensity band in the lower frequency region at 1838 and 1846 cm^−1^, respectively. It should be noted that ν(B-H_bridge_) stretching vibrations in the low-frequency region at 1700-2000 cm^−1^ are rarely observed in the IR spectra of M(η^1^-BH_4_) complexes, but as a rule always present in the spectra of M(η^2^-BH_4_) complexes [44]. The bands of bending vibrations δ(BH) in the spectra of complexes **3a**, **3b** are observed at 1054 and 1060 cm^−1^, respectively, that is in the frequency range characteristic rather for the η^1^-bound BH_4_ ligand [39,44].

## 3. Materials and Methods 

### 3.1. General Considerations

All synthetic work was performed under a purified argon atmosphere using standard Schlenk techniques. The solvents were dried and degassed by standard methods under an argon atmosphere. Deuterated solvents were freshly distilled under argon prior to use. The starting compounds 1,3-bis(di-*tert*-butylphosphinomethyl)-1′-(trifluoromethyl)-2′,3′,4′,5′-(tetramethyl)-ruthenocene (**1**) [37] and {2,5-bis(di-*tert*-butylphosphinomethyl)-1′,2′,3′,4′,5′-(pentamethyl)-ruthenocen-1-yl}palladium chloride (**2b**) [17] were prepared by known procedures.

The ^1^H, ^19^F, ^11^B{^1^H} NMR spectra were recorded on a Bruker Avance 400 spectrometer. ^1^H chemical shifts are reported in ppm downfield to TMS using the residual signals of the solvent (CDCl_3_, δ 7.26; C_6_D_6_, δ 7.16) as the internal standard. The ^19^F and ^11^B{^1^H} NMR spectra are given on the δ scale, the chemical shifts were measured relative to CFCl_3_ and BF_3_·Et_2_O, respectively, as external standards. ^31^P{^1^H} NMR spectra were recorded on Bruker Avance 300, or Bruker Avance 400 spectrometers and are reported in ppm using 85% H_3_PO_4_ as external standard. ^13^C{^1^H} NMR spectra were recorded on a Bruker Avance 600 spectrometer with CDCl_3_ (δ 77.16) or C_6_D_6_ (δ 128.06) as the internal reference. FTIR spectra were measured on Shimadzu IR Prestige 21 FTIR spectrometer. Elemental analysis was performed at the Laboratory of Microanalysis of INEOS RAS. Single crystals of **3a** and **3b** were obtained by slow crystallization from toluene/hexane mixture. 

### 3.2. Crystallographic Data

Crystals of **3a** (C_33_H_58_BF_3_P_2_PdRu, M = 792.01) are monoclinic, space group P2_1_/c, at 120 K: a = 18.5494(6), b = 12.0953(4), c = 15.8197(5) Å, β = 90.2560(10)°, V = 3549.3(2) Å^3^, Z = 4 (Z’ = 1), d_calc_ = 1.482 g·cm^−3^, μ(MoKα) = 10.59 cm^−1^, F(000) = 1632. Crystals of **3b** (C_33_H_61_BP_2_PdRu, M = 738.03) are orthorhombic, space group P2_1_2_1_2_1_, at 120 K: a = 11.3765(5), b = 15.0316(6), c = 21.0022(9) Å, V = 3591.5(3) Å^3^, Z = 4 (Z’ = 1), d_calc_ = 1.365 g·cm^−3^, μ(MoKα) = 10.30 cm^−1^, F(000) = 1536. Intensities of 72725 and 36743 reflections were measured with a Bruker APEX2 DUO CCD diffractometer [λ(MoKα) = 0.71073 Å, ω-scans, 2θ < 58°] for **3a** and **3b**, respectively; 9442 and 9578 independent reflections [R_int_ 0.0522 and 0.0518] were used in a further refinement. Using Olex2 [45], the structures were solved with the ShelXT structure solution program [46] using Intrinsic Phasing and refined with the XL refinement package [47] using Least-Squares minimization. Hydrogen atoms of the BH groups were located from difference Fourier synthesis and refined freely in the isotropic approximation. Positions of all other atoms were calculated, and they were refined within the riding model. For **3a**, the refinement converged to wR2 = 0.0535 and GOF = 1.023 for all the independent reflections (R1 = 0.0235 was calculated against F for 8034 observed reflections with I > 2σ(I)). For **3b**, the refinement converged to wR2 = 0.0729 and GOF = 1.009 for all the independent reflections (R1 = 0.0394 was calculated against F for 8230 observed reflections with I>2σ(I)). CCDC 1993081 and 1993082 contain the supplementary crystallographic information for **3a** and **3b**, respectively. Crystal data and structure refinement parameters for **3a**, **3b** are summarized in Table S1.

### 3.3. Synthesis

#### 3.3.1. Preparation of {2,5-Bis(di-tert-butylphosphinomethyl)-1′-(trifluoromethyl)-2′,3′,4′,5′-(tetramethyl)ruthenocen-1-yl]palladium(II) chloride PdCl[{2,5-(^t^Bu_2_PCH_2_)_2_C_5_H_2_}Ru(C_5_Me_4_CF_3_)] (**2a**)

PdCl_2_(PhCN)_2_ (270 mg, 0.703 mmol) was added to a suspension of bisphosphine **1** (470 mg, 0.700 mmol) in 2-methoxyethanol (20 mL). The mixture was refluxed with stirring for 3 h, then triethylamine (0.20 mL, 1.45 mmol) was rapidly added to the boiling solution using a syringe and the mixture was refluxed for additional 2 h. After cooling the resulting solution was diluted with dichloromethane (1:1) and filtered through celite. Then the solvents were removed in vacuo, and the residue was purified on an alumina column (eluent hexane- dichloromethane (2:1)). Recrystallization of the residue from ethanol gave pale-yellow solid of the product. Yield: 175 mg (31%). ^1^H NMR (400.13 MHz, CDCl_3_, 294 K): 1.33 (vt, 18H, *J*_HP_ = 13.1, C(CH_3_)_3_), 1.47 (vt, 18H, *J*_HP_ = 14.6, C(CH_3_)_3_), 1.77 (s, 6H, 3,4-C_5_(CH_3_)_2_(CH_3_)_2_CF_3_), 1.89 (br. m, 6H, 2,5-C_5_(CH_3_)_2_(CH_3_)_2_CF_3_), 2.45 (dvt, 2H, *J*_HH_ = 16.6, *J*_HP_ = 6.3, CH_A_H_B_P), 2.51 (dvt, 2H, *J*_HH_ = 16.6, *J*_HP_ = 8.7, CH_A_H_B_P), 4.22 (s, 2H, C_5_H_2_). ^31^P{^1^H} NMR (161.98 MHz, CDCl_3_, 294 K): 82.07 (s, 2P). ^19^F NMR (376.50 MHz, CDCl_3_, 294 K): -52.74 (s, 3F, CF_3_). ^13^C{^1^H} NMR (150.93 MHz, CDCl_3_, 293 K): 10.61 (s, 2C, 3,4-C_5_(CH_3_)_2_(CH_3_)_2_CF_3_), 11.23 (q, 2C, *J*_CF_ = 2.3, 2,5-C_5_(CH_3_)_2_(CH_3_)_2_CF_3_), 22.68 (vt, 2C, *J*_CP_ = 19.5, CH_2_), 29.58 (vt, 6C, *J*_CP_ = 5.7, C(CH_3_)_3_), 29.75 (vt, 6C, *J*_CP_ = 7.3, C(CH_3_)_3_), 34.36 (vt, 2C, *J*_CP_ = 13.7, C(CH_3_)_3_), 35.79 (vt, 2C, *J*_CP_ = 11.2, C(CH_3_)_3_), 71.48 (vt, 2C, *J*_CP_ = 16.5, 3,4-C_5_H_2_), 76.77 (q, 1C, *J*_CF_ = 35.5, C-CF_3_), 82.50 (s, 2C, C_5_(CH_3_)_4_CF_3_), 87.43 (s, 2C, C_5_(CH_3_)_4_CF_3_), 97.26 (vt, 2C, *J*_CP_ = 29.3, 2,5-C_5_H_2_), 118.98 (s, 1C, 1-C_5_H_2_), 128.27 (q, 1C, *J*_CF_ = 270.0, CF_3_). Anal. Calc. for C_33_H_54_F_3_ClP_2_PdRu (Mr = 812.75): C, 48.76; H, 6.71; P, 7.62; Cl, 4.36%. Found: C, 48.54; H, 6.82; P, 7.49; Cl, 4.48%.

#### 3.3.2. General Procedure for Preparation of Palladium Tetrahydroborate Complexes **3a** and **3b**

NaBH_4_ (100 mg, 2.63 mmol) was added to a solution of complexes **2a** or **2b** (110–120 mg) in 20 mL of ethanol-benzene (10:1) mixture at room temperature. The mixture was refluxed for 8 h, adding 50 mg (1.32 mmol) of NaBH_4_ to the mixture every hour. The reaction mixture was then cooled and evaporated in vacuo to a minimum volume. Distilled water (20 mL) was added to the residue, the resulting suspension was stirred at room temperature for 1 h. The reaction mass was extracted (4 × 10 mL) with hexane-benzene (3:1). The solvents were evaporated in vacuo. Recrystallization of the residue from toluene-hexane (1:1) mixture gave solid products (**3a** or **3b**), which were additionally dried in vacuo at room temperature.

##### Preparation of {2,5-Bis(di-tert-butylphosphinomethyl)-1′-(trifluoromethyl)-2′,3′,4′,5′-(tetramethyl)-ruthenocen-1-yl]palladium(II) tetrahydroborate Pd(BH_4_)[{2,5-(^t^Bu_2_PCH_2_)_2_C_5_H_2_}Ru(C_5_Me_4_CF_3_)] (**3a**)

Complex **3a** obtained from 118 mg (0.145 mol) of complex **2a** as a yellow powder. Yield: 104 mg (90%). ^1^H NMR (400.13 MHz, C_6_D_6_, 293 K): 0.13 (br. m, 4H, BH_4_), 1.15 (vt, 18H, *J*_HP_ = 13.4, C(CH_3_)_3_), 1.40 (vt, 18H, *J*_HP_ = 14.4, C(CH_3_)_3_), 1.66 (s, 6H, 3,4-C_5_(CH_3_)_2_(CH_3_)_2_CF_3_), 1.95 (br. m, 6H, 2,5-C_5_(CH_3_)_2_(CH_3_)_2_CF_3_), 2.08 (dvt, 2H, *J*_HH_ = 16.6, *J*_HP_ = 9.2, CH_A_H_B_P), 2.32 (dvt, 2H, *J*_HH_ = 16.6, *J*_HP_ = 5.9, CH_A_H_B_P), 4.25 (s, 2H, C_5_H_2_). ^11^B{^1^H} (128.38 MHz, C_6_D_6_, 295 K): −34.54 (br.s, 1B, BH_4_). ^31^P{^1^H} NMR (121.49 MHz, C_6_D_6_, 293 K): 87.44 (s, 2P). ^19^F NMR (376.50 MHz, C_6_D_6_, 295 K): −52.05 (s, 3F, CF_3_). ^13^C{^1^H} NMR (150.93 MHz, C_6_D_6_, 294 K): 10.57 (s, 2C, 3,4-C_5_(CH_3_)_2_(CH_3_)_2_CF_3_), 11.44 (br. m, 2C, 2,5-C_5_(CH_3_)_2_(CH_3_)_2_CF_3_), 23.36 (vt, 2C, *J*_CP_ = 20.3, CH_2_), 29.55 (vt, 6C, *J*_CP_ = 7.1, C(CH_3_)_3_), 29.63 (vt, 6C, *J*_CP_ = 6.0, C(CH_3_)_3_), 34.70 (vt, 2C, *J*_CP_ = 14.4, C(CH_3_)_3_), 35.27 (vt, 2C, *J*_CP_ = 11.4, C(CH_3_)_3_), 71.43 (vt, 2C, *J*_CP_ = 16.5, 3,4-C_5_H_2_), 77.03 (q, 1C, *J*_CF_ = 35.3, C-CF_3_), 82.65 (s, 2C, C_5_(CH_3_)_4_CF_3_), 87.45 (s, 2C, C_5_(CH_3_)_4_CF_3_), 97.82 (vt, 2C, *J*_CP_ = 28.8, 2,5-C_5_H_2_), 121. 77 (s, 1C, 1-C_5_H_2_), 129.22 (q, 1C, *J*_CF_ = 269.9, CF_3_). FTIR (KBr pellet, ν in cм^−1^): 2388 (s, B-H_t_), 2363 (s, B-H_t_), 2296 (s, B-H_t_), 2019 (br. w, B-H_br_-Pd), 1838 (br. s, B-H_br_-Pd), 1054 (s, δ_BH3_). FTIR (CH_2_Cl_2_, ν in cm^−1^): 2376 (s(sh), B-H_t_), 2289 (s, B-H_t_), 2019 (br. w, B-H_br_-Pd), 1840 (s, B-H_br_-Pd), 1062 (s, δ_BH3_). Anal. Calc. for C_33_H_58_BF_3_P_2_PdRu (M_r_ = 792.06): C, 50.04; H, 7.38%. Found: C, 50.29; H, 7.48%.

##### Preparation of {2,5-Bis(di-tert-butylphosphinomethyl)-1′,2′,3′,4′,5′-(pentamethyl)ruthenocen-1-yl]palladium(II) tetrahydroborate Pd(BH_4_)[{2,5-(^t^Bu_2_PCH_2_)_2_C_5_H_2_}Ru(C_5_Me_5_)] (**3b**)

Complex **3b** obtained from 114 mg (0.150 mol) of complex **2b** as a yellow powder. Yield: 103 mg (93%). ^1^H NMR (400.13 MHz, C_6_D_6_, 295 K): 0.21 (br.m, 4H, BH_4_), 1.20 (vt, 18H, *J*_HP_ = 13.0, C(CH_3_)_3_), 1.47 (vt, 18H, *J*_HP_ = 14.3, C(CH_3_)_3_), 1.84 (s, 15H, C_5_(CH_3_)_5_), 2.15 (dvt, 2H, *J*_HH_ = 16.5, *J*_HP_ = 9.1, CH_A_H_B_P), 2.38 (dvt, 2H, *J*_HH_ = 16.5, *J*_HP_ = 5.9, CH_A_H_B_P), 3.95 (s, 2H, C_5_H_2_). ^11^B{^1^H} (128.38 MHz, C_6_D_6_, 295 K): −34.73 (br.s, 1B, BH_4_). ^31^P{^1^H} NMR (161.98 MHz, C_6_D_6_, 295 K): 87.07 (s, 2P). ^13^C{^1^H} NMR (150.93 MHz, C_6_D_6_, 293 K): 11.49 (s, 5C, C_5_(CH_3_)_5_), 23.42 (vt, 2C, *J*_CP_ = 20.4, CH_2_), 29.71 (m, 12C, C(CH_3_)_3_), 34.67 (vt, 2C, *J*_CP_ = 14.3, C(CH_3_)_3_), 35.22 (vt, 2C, *J*_CP_ = 11.1, C(CH_3_)_3_), 71.43 (vt, 2C, *J*_CP_ = 16.9, 3,4-C_5_H_2_), 83.99 (s, 5C, C_5_(CH_3_)_5_), 95.84 (vt, 2C, *J*_CP_ = 29.1, 2,5-C_5_H_2_), 120. 55 (s, 1C, 1-C_5_H_2_). FTIR (KBr pellet, ν in cм^-1^): 2374 (s(sh), B-H_t_), 2291 (s, B-H_t_), 2025 (br.w, B-H_br_-Pd), 1851 (s, B-H_br_-Pd), 1060 (s, δ_BH3_). FTIR (CH_2_Cl_2_, ν in cm^−1^): 2372 (s(sh), B-H_t_), 2287 (s, B-H_t_), 2008 (br. w, B-H_br_-Pd), 1841 (s, B-H_br_-Pd), 1062 (s, δ_BH3_). Anal. Calc. for C_33_H_61_BP_2_PdRu (M_r_ = 738.09): C, 53.70; H, 8.33; P, 8.39%. Found: C, 53.84; H, 8.38; P, 8.13%.

## 4. Conclusions

Thus, our work demonstrated that the introduction of the bulky C_5_Me_4_CF_3_ (Cp^F^) and C_5_Me_5_ (Cp*) ligands in the sandwich scaffold of the ruthenocene-based PCP^tBu^ pincer palladium complexes does not preclude the formation of the corresponding palladium tetrahydroborate pincer complexes. The two novel sterically loaded pincer palladium tetrahydroborates Rc^F^[PCP^tBu^]Pd(BH_4_) (**3a**) and Rc*[PCP^tBu^]Pd(BH_4_) (**3b**) were synthesized and fully characterized by X-ray, NMR, and FTIR techniques. The X-ray diffraction study of **3a** and **3b** revealed that an increase of the steric bulk of non-metalated cyclopentadienyl ring in **3a** and **3b** relative to non-substituted Rc[PCP^tBu^]Pd(BH_4_) analogue (**3c**) pushes palladium atom from the middle plane of the metalated Cp ring in the direction opposite to the ruthenium atom. This displacement increases in the order **3c** < **3b** < **3a** following the order of the Cp-ring steric volume increase. The analysis of both X-ray and IR data suggests that BH_4_ ligand in both palladium tetrahydroborates **3a** and **3b** has the mixed coordination mode η^1,2^ similar to that in **3c** in which the primary Pd-H contact of 1.82–1.87Å is accompanied by the secondary interaction Pd···H of 2.34–2.54Å. Pd⋯B distances decrease in the order: Rc[PCP^tBu^]Pd(BH_4_) > Rc*[PCP^tBu^]Pd(BH_4_) > Rc^F^[PCP^tBu^]Pd(BH_4_) that suggests the increase in the strength of the BH_4_ bond with the palladium atom that appears to be affected by both steric and electronic properties of the ruthenocene moiety.

## Figures and Tables

**Table 1:** Selected FTIR data for complexes **3a**–**3c** (band positions in cm^−1^).

	3a(KBr)	3b(KBr)	3c(KBr) [36]	3a(CH_2_Cl_2_)	3b(CH_2_Cl_2_)
ν^as^(B-H_term_)	2388, 2363	2374	2364	2376	2372
ν^s^(B-H_term_)	2296	2291	2292	2289	2287
ν_1_(B-H_bridge_)	2019	2025	1993	2019	2008
ν_2_(B-H_bridge_)	1838	1846	1840	1840	1841
δ(BH)	1054	1060		1062	1062

## Supplementary Materials

The following are available online, Table S1: Crystal data and structure refinement parameters for **3a**, **3b**. Figure S1: ^1^H NMR spectrum (400.13 MHz) of **2a** in CDCl_3_. Figure S2: ^31^P{^1^H} NMR spectrum (161.98 MHz) of **2a** in CDCl_3_. Figure S3: ^19^F NMR spectrum (376.50 MHz) of **2a** in CDCl_3_. Figure S4: ^13^C{^1^H} NMR spectrum (150.93 MHz) of **2a** in CDCl_3_. Figure S5: ^1^H NMR spectrum (400.13 MHz) of **3a** in C_6_D_6_. Figure S6: ^11^B{^1^H} NMR spectrum (128.38 MHz) of **3a** in C_6_D_6_. Figure S7: ^31^P{^1^H} NMR spectrum (121.49 MHz) of **3a** in C_6_D_6_. Figure S8: ^19^F NMR spectrum (376.50 MHz) of **3a** in C_6_D_6_. Figure S9: ^13^C{^1^H} NMR spectrum (150.93 MHz) of **3a** in C_6_D_6_. Figure S10: ^1^H NMR spectrum (400.13 MHz) of **3b** in C_6_D_6_. Figure S11: ^11^B{^1^H} NMR spectrum (128.38 MHz) of **3b** in C_6_D_6_. Figure S12: ^31^P{^1^H} NMR spectrum (161.98 MHz) of **3b** in C_6_D_6_. Figure S13: ^13^C{^1^H} NMR spectrum (150.93 MHz) of **3b** in C_6_D_6_. Figure S14: FTIR spectra of **3a** in KBr pellet. Figure S15: FTIR spectra of **3b** in KBr pellet. Figure S16: FTIR spectra of **3a** in the CH_2_Cl_2_ solution. Figure S17: FTIR spectra of **3b** in the CH_2_Cl_2_ solution.
