# Supplementary material for: Steric and Electronic Effect of Cp-Substituents on the Structure of the Ruthenocene Based Pincer Palladium Borohydrides
Source: Molecules. 2020 May 9;25(9):2236. doi: 10.3390/molecules25092236 (PMC7248887; doi:10.3390/molecules25092236)

Supplementary Materials

Steric and electronic effect of Cp-substituents on the structure of the ruthenocene based palladium pincer borohydrides

Sergey V. Safronov [1,*], Yulia V. Nelubina [1], Oleg A. Filippov [1],
Irina G. Barakovskaya [1], Natalia V. Belkova [1,*] and Elena S. Shubina [1]

[1] A. N. Nesmeyanov Institute of Organoelement Compounds, Russian Academy of Sciences, 28 Vavilova Str., Moscow 119991, Russia;

* Correspondence: nataliabelk@ineos.ac.ru (N.V.B.); sergiosfr-hcc@mail.ru (S.V.S.)

Table of content

Table S1. Crystal data and structure refinement parameters for **3a**, **3b**. S2

Figure S1. ^{1}H NMR spectrum (400.13 MHz) of **2a** in $CDCl_3$. S3

Figure S2. $^{31}P\{^{1}H\}$ NMR spectrum (161.98 MHz) of **2a** in $CDCl_3$. S3

Figure S3. ^{19}F NMR spectrum (376.50 MHz) of **2a** in $CDCl_3$. S4

Figure S4. $^{13}C\{^{1}H\}$ NMR spectrum (150.93 MHz) of **2a** in $CDCl_3$. S4

Figure S5. ^{1}H NMR spectrum (400.13 MHz) of **3a** in C_6D_6. S5

Figure S6. $^{11}B\{^{1}H\}$ NMR spectrum (128.38 MHz) of **3a** in C_6D_6. S5

Figure S7. $^{31}P\{^{1}H\}$ NMR spectrum (121.49 MHz) of **3a** in C_6D_6. S6

Figure S8. ^{19}F NMR spectrum (376.50 MHz) of **3a** in C_6D_6. S6

Figure S9. $^{13}C\{^{1}H\}$ NMR spectrum (150.93 MHz) of **3a** in C_6D_6. S7

Figure S10. ^{1}H NMR spectrum (400.13 MHz) of **3b** in C_6D_6. S7

Figure S11. $^{11}B\{^{1}H\}$ NMR spectrum (128.38 MHz) of **3b** in C_6D_6. S8

Figure S12. $^{31}P\{^{1}H\}$ NMR spectrum (161.98 MHz) of **3b** in C_6D_6. S8

Figure S13. $^{13}C\{^{1}H\}$ NMR spectrum (150.93 MHz) of **3b** in C_6D_6. S9

Figure S14. FTIR spectra of **3a** in KBr pellet. S10

Figure S15. FTIR spectra of **3b** in KBr pellet. S10

Figure S16. FTIR spectra of **3a** in the CH_2Cl_2 solution. S11

Figure S17. FTIR spectra of **3b** in the CH_2Cl_2 solution. S11

Table S1. Crystal data and structure refinement parameters for **3a**, **3b**.

	**3a**	**3b**
Empirical formula	$C_{33}H_{58}BF_3P_2PdRu$	$C_{33}H_{61}BP_2PdRu$
Formula weight	792.01	738.03
Crystal system	Monoclinic	Orthorhombic
Space group	$P2_1/c$	$P2_12_12_1$
a, Å	18.5494(6)	11.3765(5)
b, Å	12.0953(4)	15.0316(6)
c, Å	15.8197(5)	21.0022(9)
α, °	90	90
β, °	90.2560(10)	90
γ, °	90	90
V, $Å^3$	3549.3(2)	3591.5(3)
Z	4	4
D_{calc} (g·cm^{-3})	1.482	1.365
m(cm^{-1})	10.59	10.30
F(000)	1632	1536
$2\Theta_{max}$, °	58	58
Reflections measured	72725	36743
Independent reflections	9442	9578
Observed reflections [I > 2s(I)]	8034	8230
R_1	0.0235	0.0394
wR_2	0.0535	0.0729
GOF	1.023	1.009

Figure S1. ^{1}H NMR spectrum (400.13 MHz) of **2a** in $CDCl_3$.

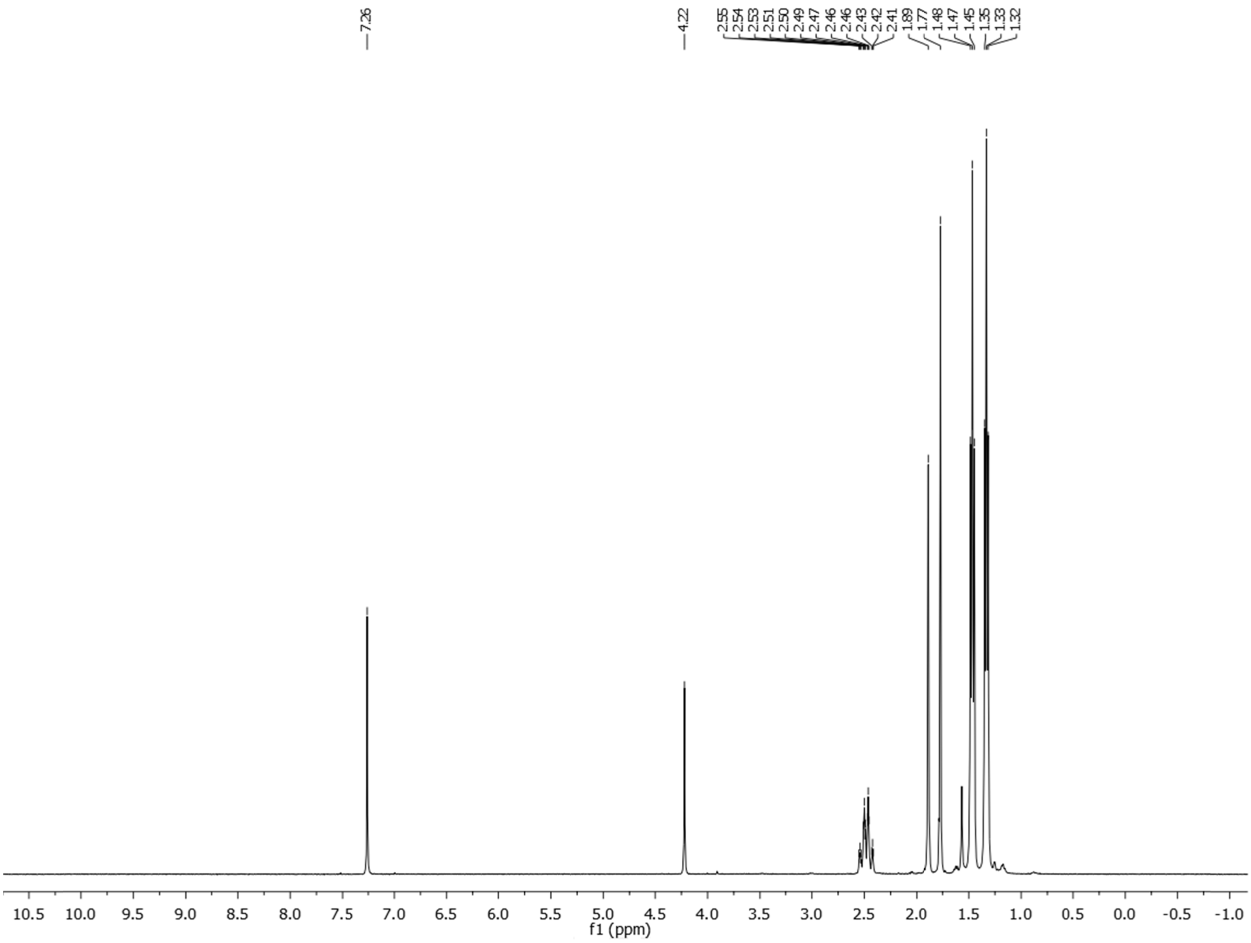

Figure S2. $^{31}P\{^{1}H\}$ NMR spectrum (161.98 MHz) of **2a** in $CDCl_3$.

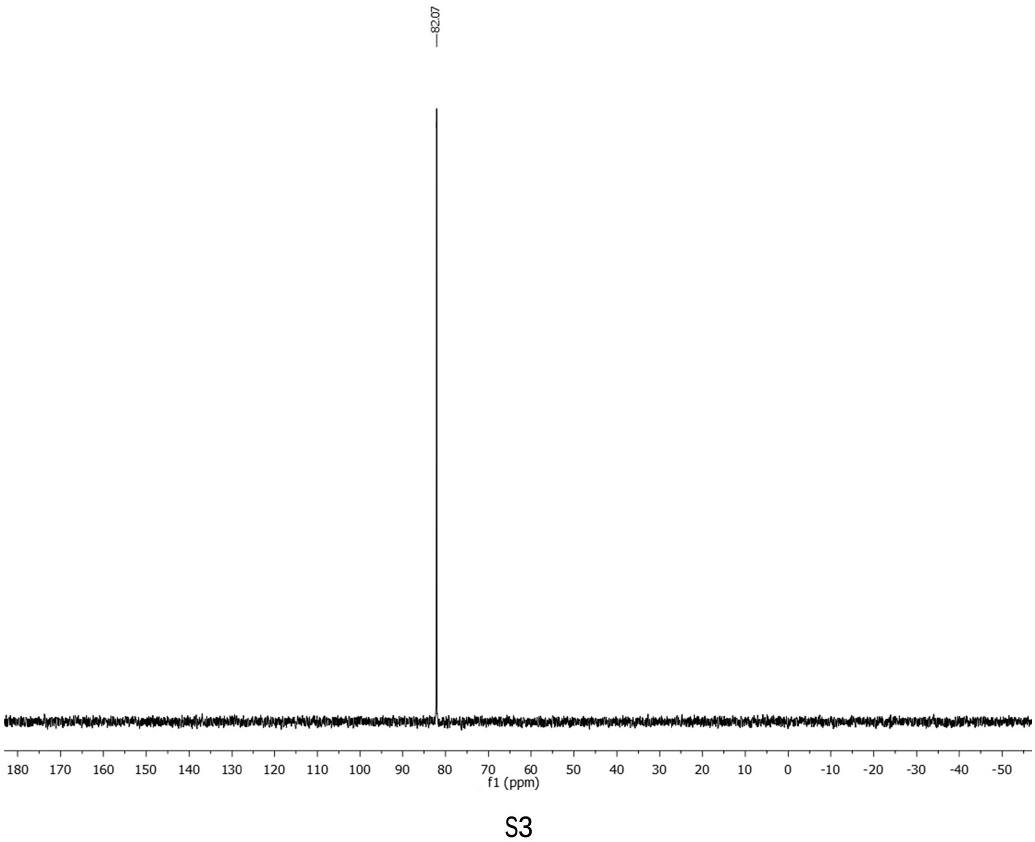

Figure S3. ^{19}F NMR spectrum (376.50 MHz) of **2a** in $CDCl_3$.

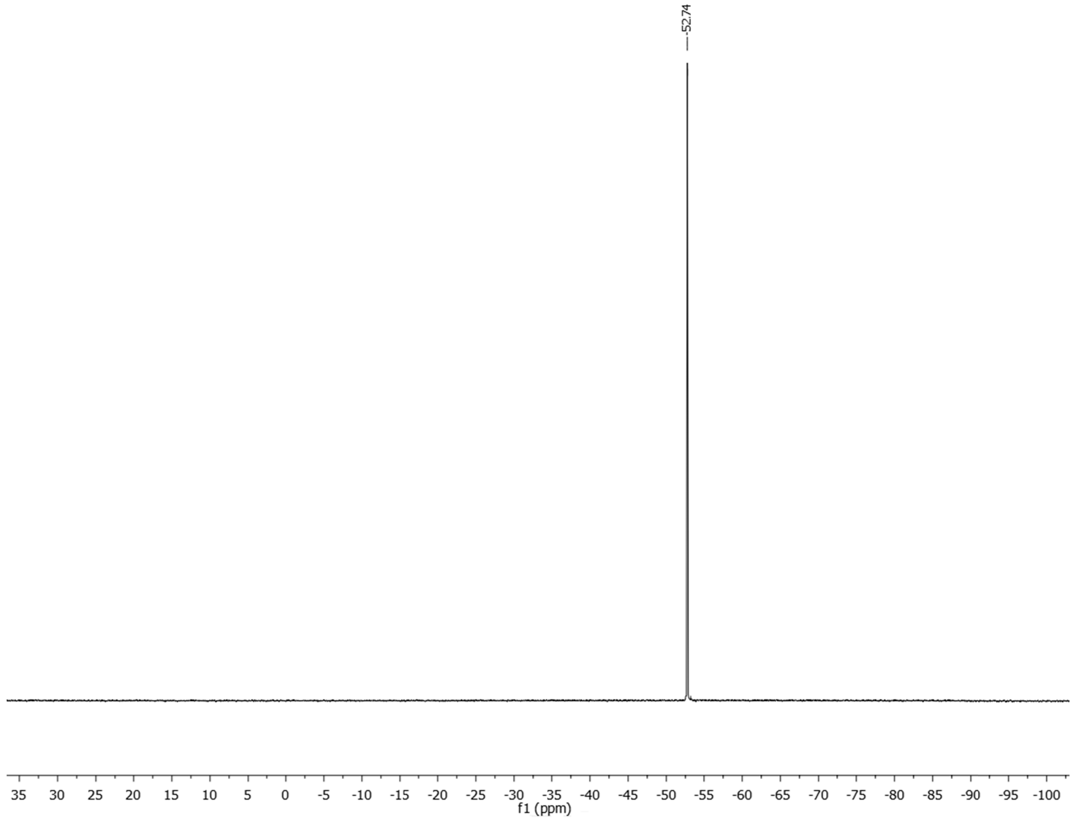

Figure S4. $^{13}C\{^{1}H\}$ NMR spectrum (150.93 MHz) of **2a** in $CDCl_3$.

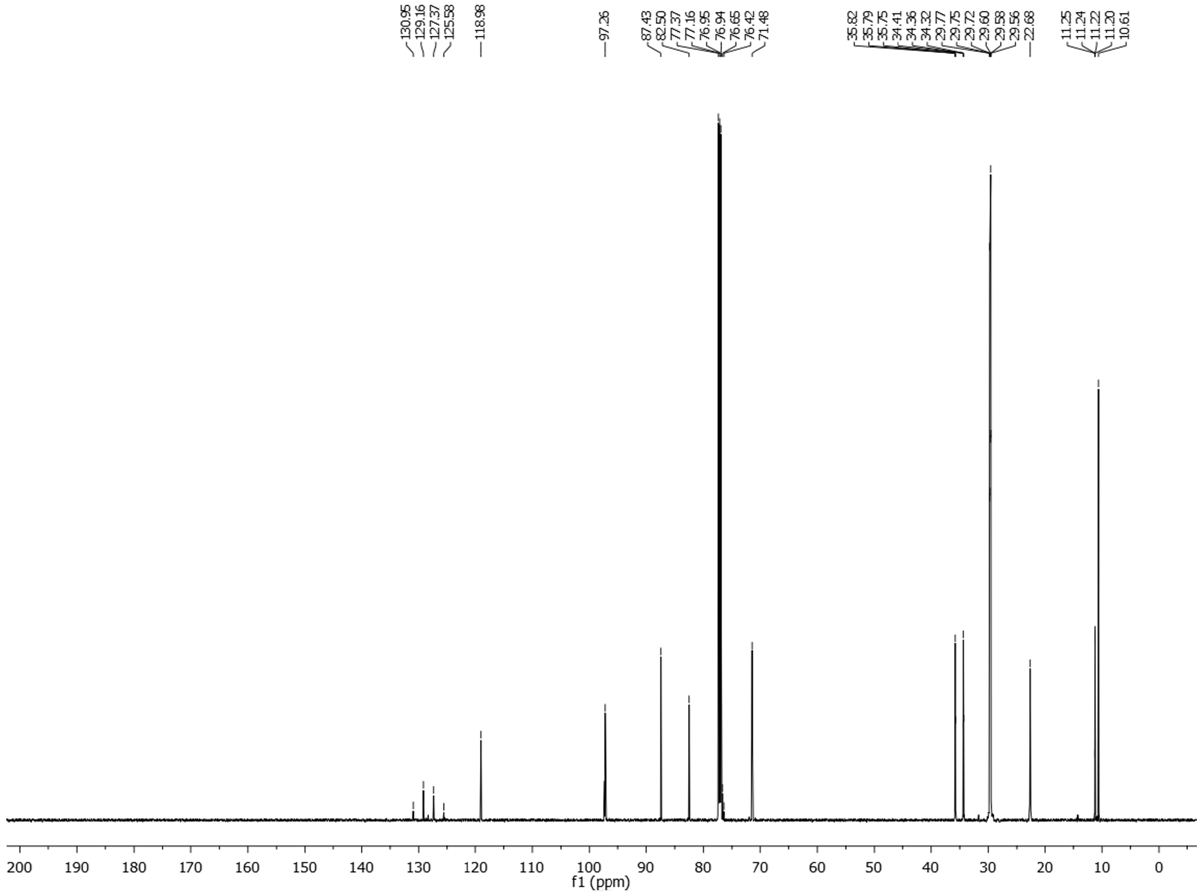

Figure S5. ^{1}H NMR spectrum (400.13 MHz) of **3a** in C_6D_6.

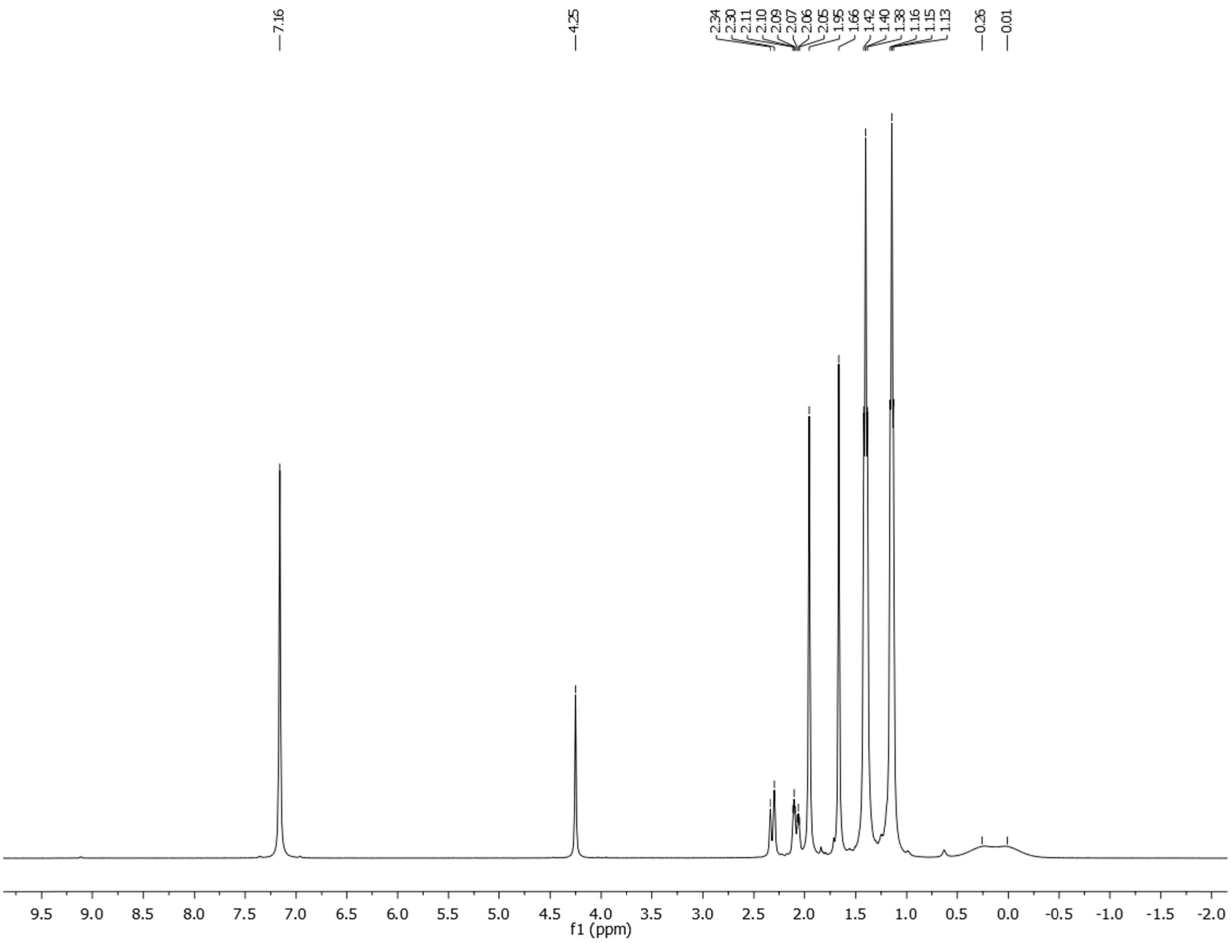

Figure S6. $^{11}B\{^{1}H\}$ NMR spectrum (128.38 MHz) of **3a** in C_6D_6.

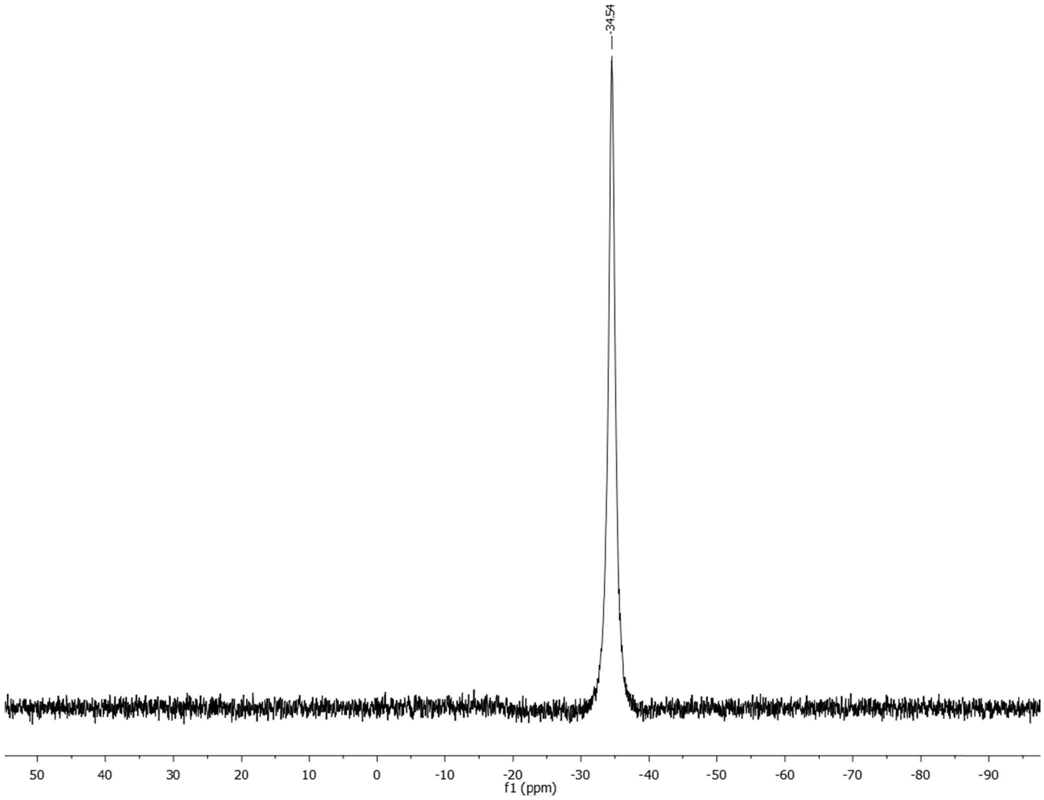

Figure S7. $^{31}P\{^1H\}$ NMR spectrum (121.49 MHz) of **3a** in C_6D_6.

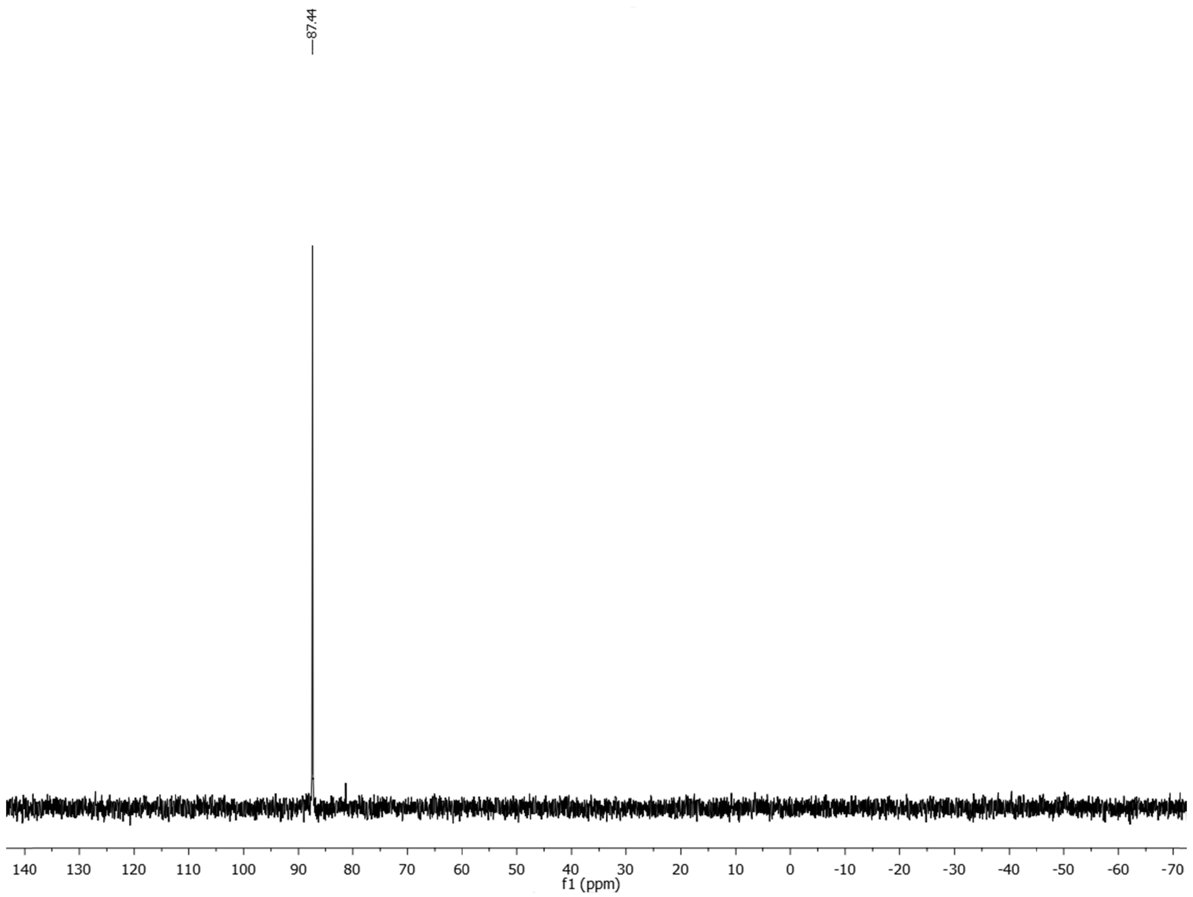

Figure S8. ^{19}F NMR spectrum (376.50 MHz) of **3a** in C_6D_6.

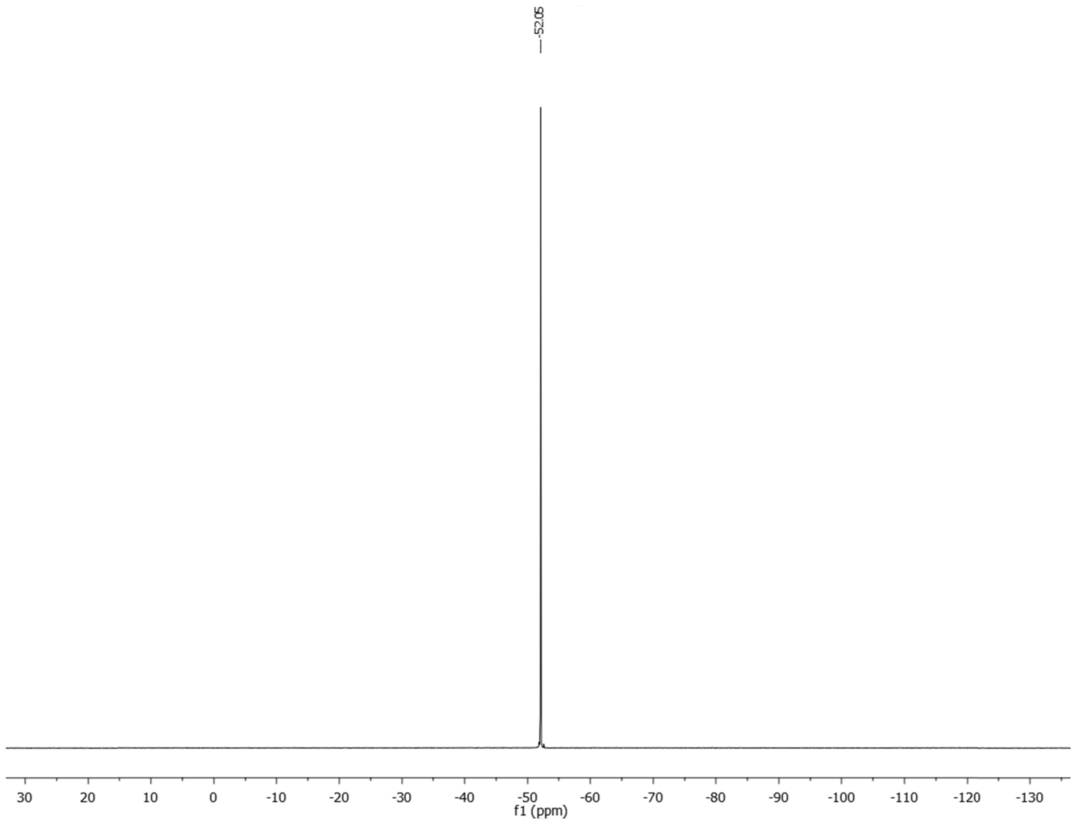

Figure S9. $^{13}C\{^{1}H\}$ NMR spectrum (150.93 MHz) of **3a** in C_6D_6.

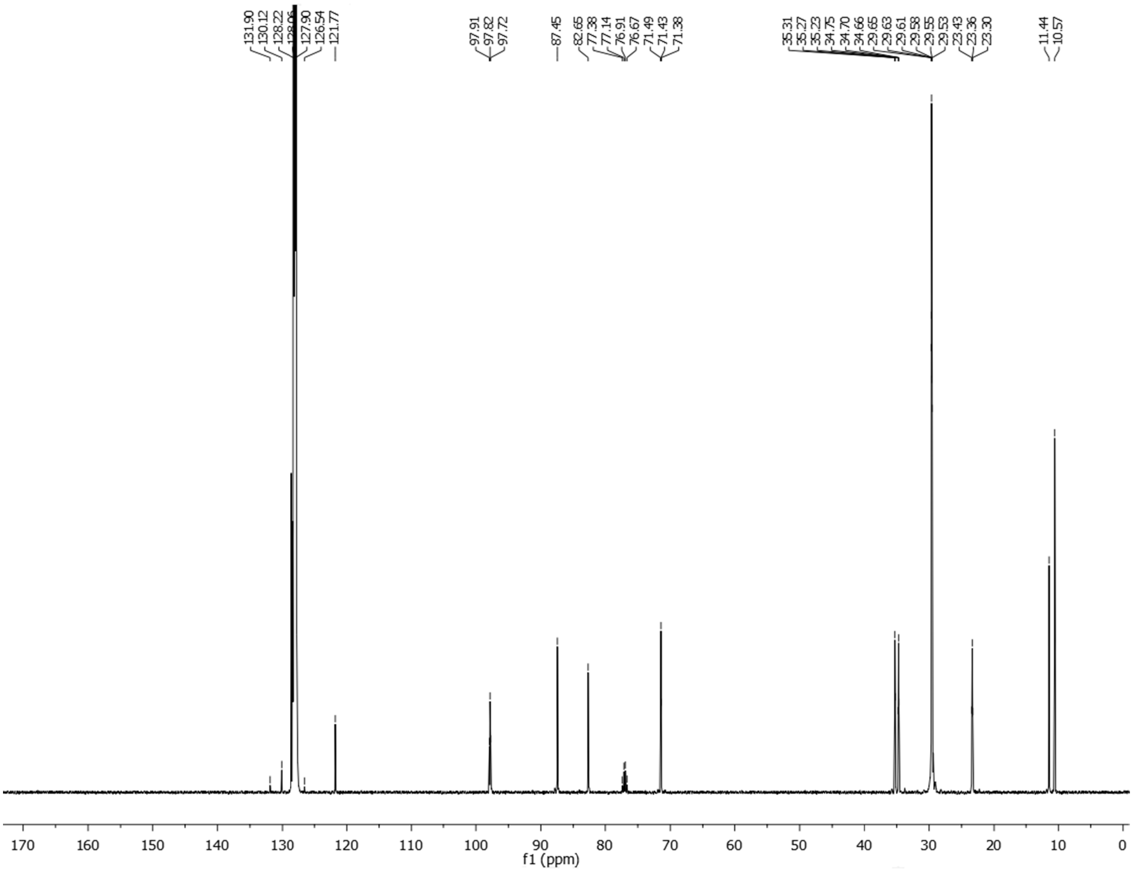

Figure S10. ^{1}H NMR spectrum (400.13 MHz) of **3b** in C_6D_6.

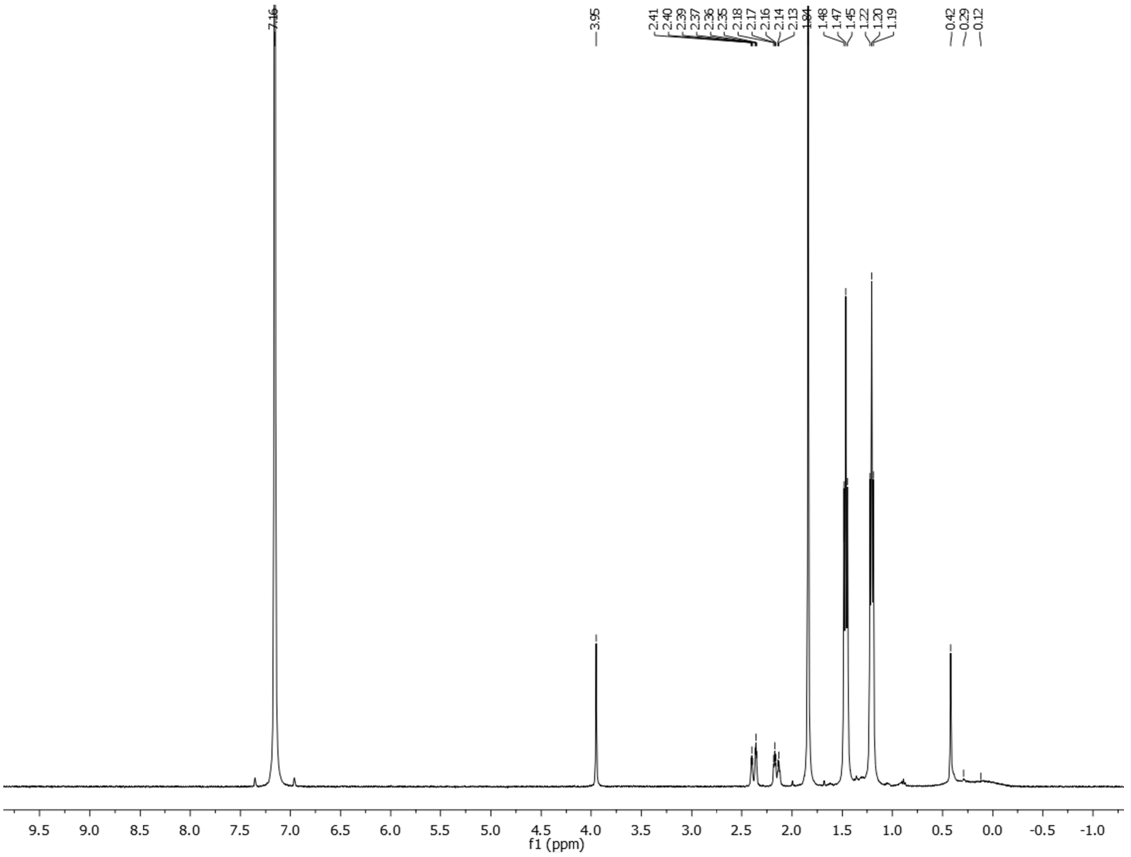

Figure S11. $^{11}B\{^{1}H\}$ NMR spectrum (128.38 MHz) of **3b** in C_6D_6.

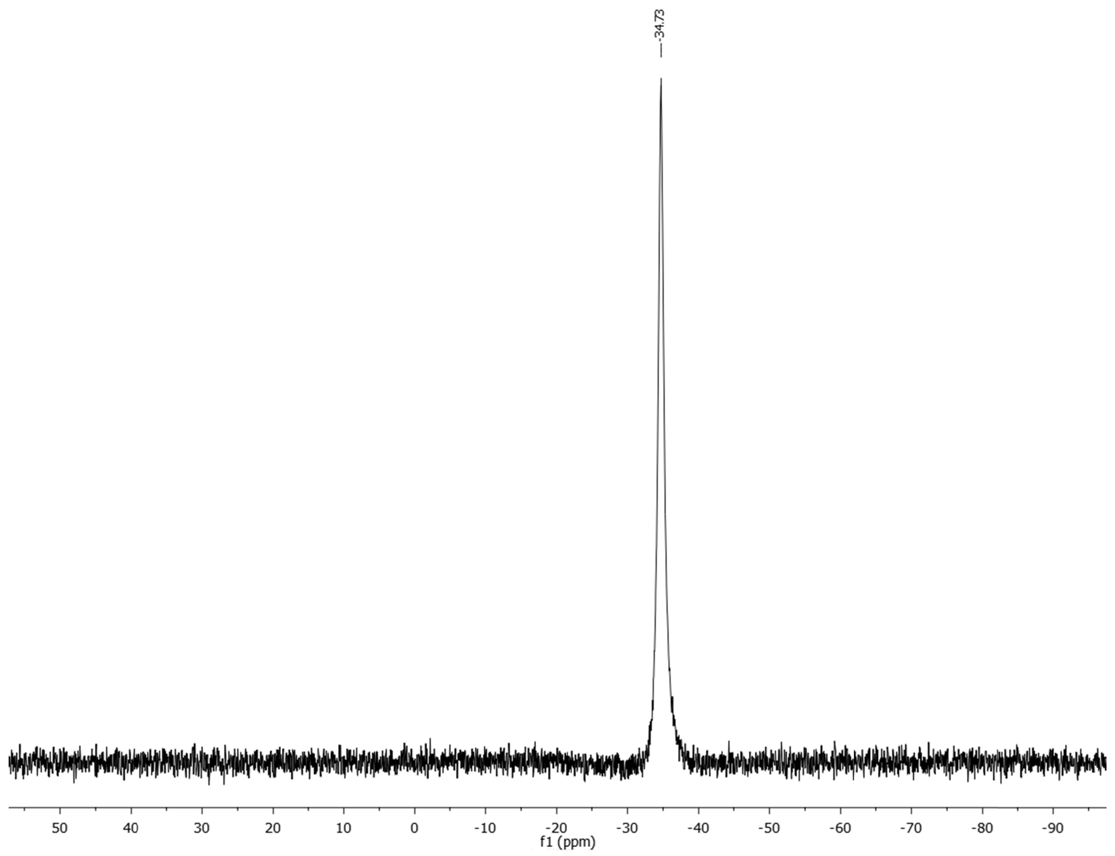

Figure S12. $^{31}P\{^{1}H\}$ NMR spectrum (161.98 MHz) of **3b** in C_6D_6.

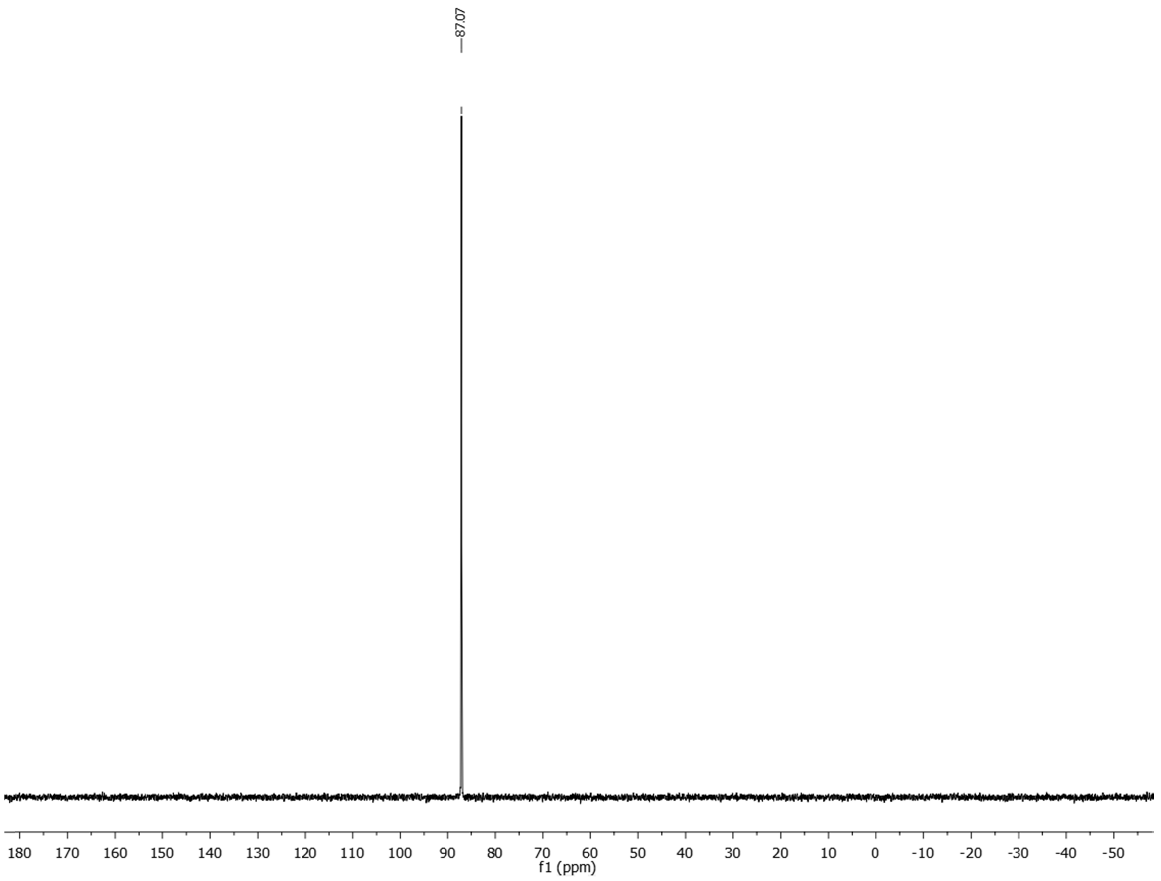

Figure S13. $^{13}C\{^1H\}$ NMR spectrum (150.93 MHz) of **3b** in C_6D_6.

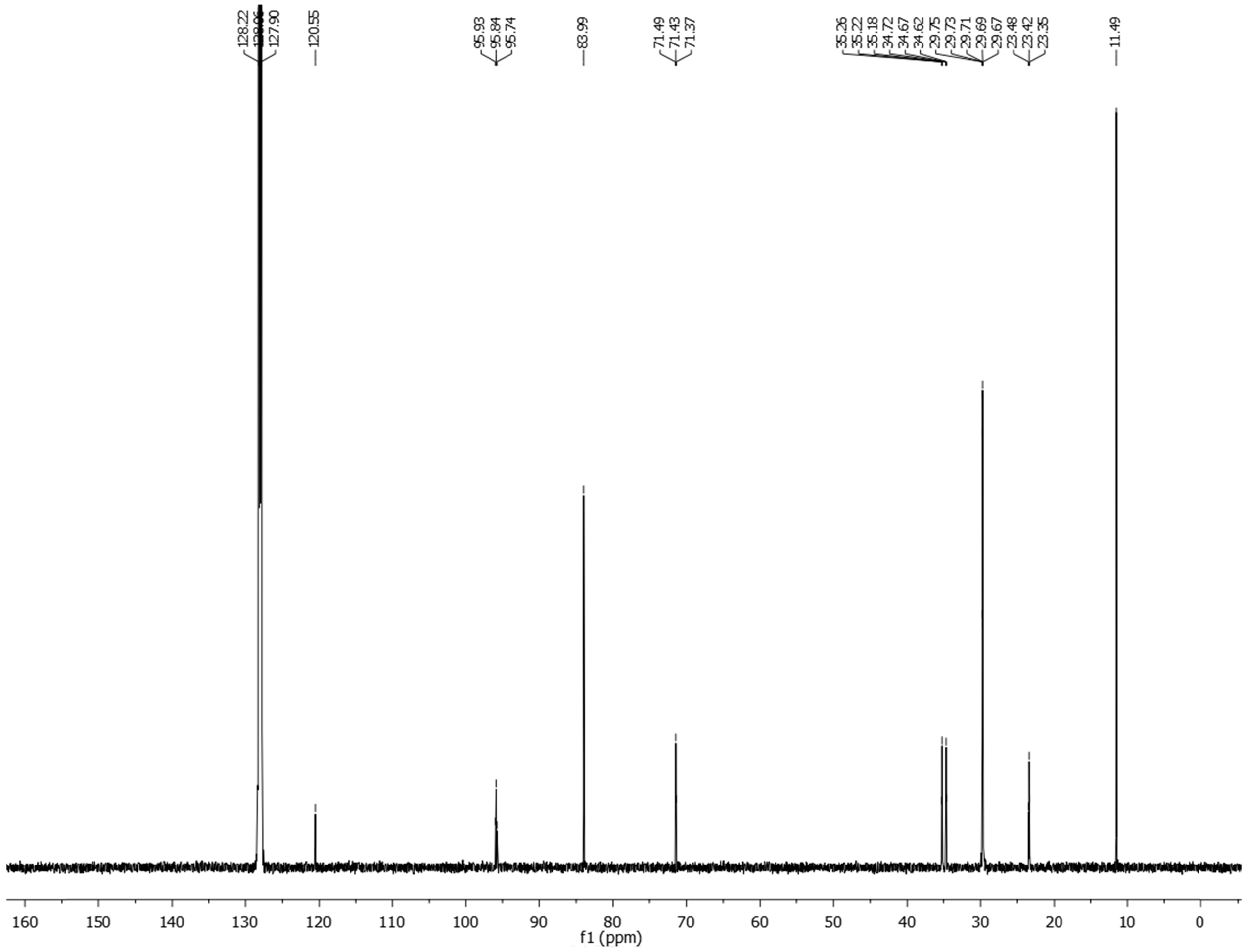

Figure S14. FTIR spectra of **3a** in KBr pellet.

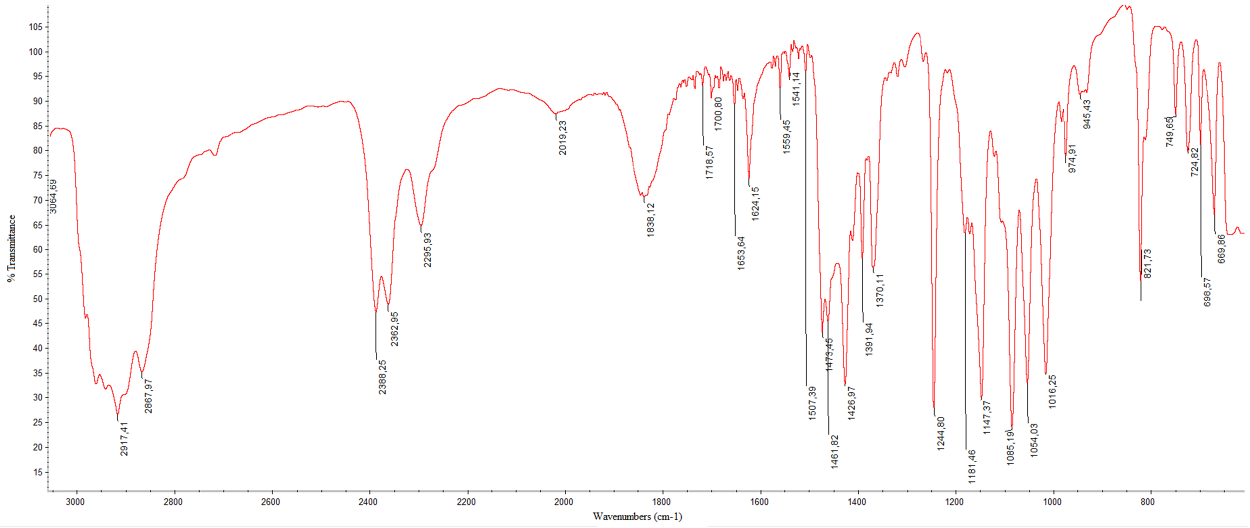

Figure S15. FTIR spectra of **3b** in KBr pellet.

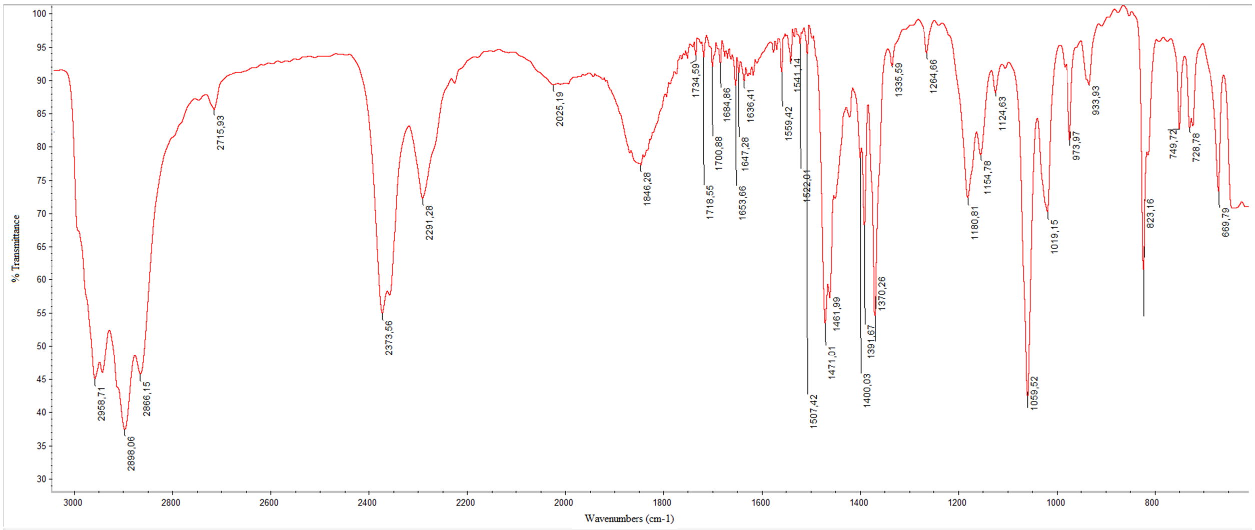

Figure S16. FTIR spectra of **3a** solution in CH_2Cl_2 (c = 0.01 M).

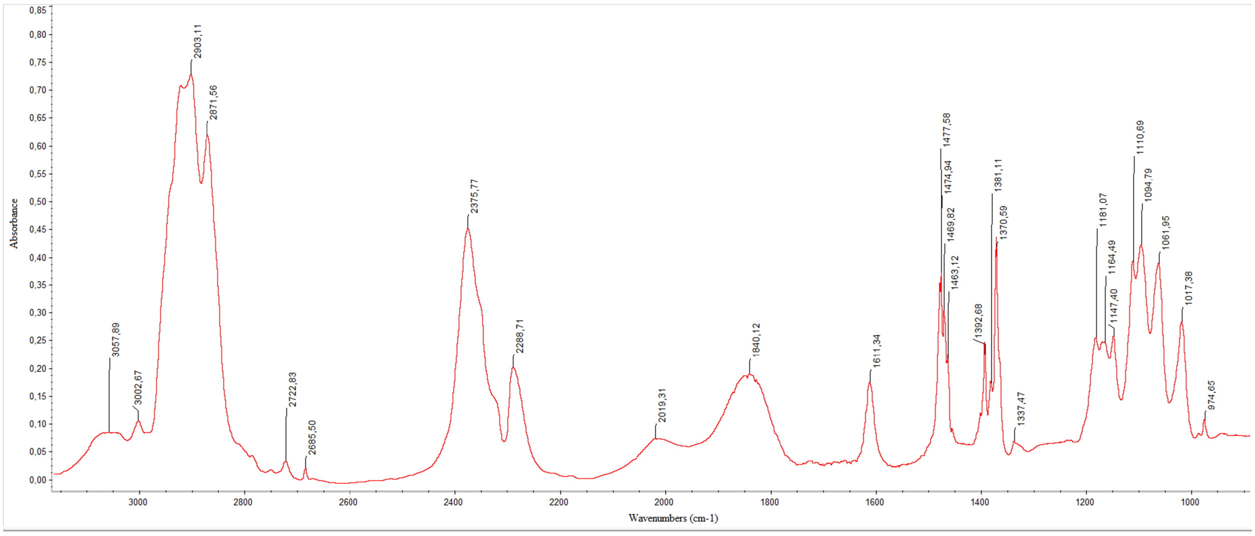

Figure S17. FTIR spectra of **3b** solution in CH_2Cl_2 (c = 0.01 M).

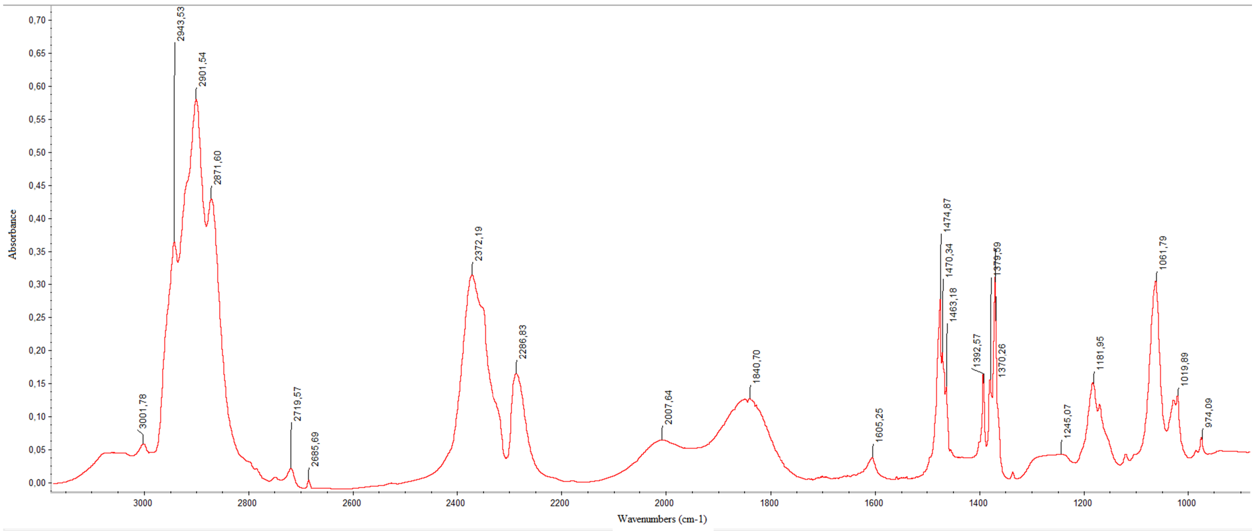

Supplement: Supplementary file 1 [file molecules-25-02236-s001.pdf]
